# Assessment of the diagnostic value of different biomarkers in relation to various stages of diabetic nephropathy in type 2 diabetic patients

**DOI:** 10.1038/s41598-017-02421-9

**Published:** 2017-06-02

**Authors:** Khalid Al-Rubeaan, Khalid Siddiqui, Mohammed A. Al-Ghonaim, Amira M. Youssef, Ahmed H. Al-Sharqawi, Dhekra AlNaqeb

**Affiliations:** 1 University Diabetes Center, College of medicine, King Saud University, Riyadh, PO Box 18397, Saudi Arabia; 2 Strategic Center for Diabetes Research, King Saud University, Riyadh, PO Box 245, Saudi Arabia; 3 College of medicine, King Khalid University Hospital, King Saud University, Riyadh, PO Box 45299, Saudi Arabia; 4 Registry Department, University Diabetes Center, King Saud University, Riyadh, PO Box 245, Saudi Arabia; 5 Research Department, University Diabetes Center, King Saud University, PO Box 245, Riyadh, Saudi Arabia

## Abstract

Albuminuria is widely used to indicate early phases of diabetic nephropathy although it is limited by the fact that structural damage might precede albumin excretion. This necessitates identifying better biomarkers that diagnose or predict diabetic nephropathy. This is a cross-sectional hospital based study recruiting type 2 diabetic patients cohort aged 35–75 years with diabetes duration of ≥10 years. Out of total eligible 467 patients, 200 patients were with normal albumin excretion, 184 patients with microalbuminuria and 83 patients with macroalbuminuria. All the patients were tested for the 22 selected biomarkers including serum, plasma and urinary markers. Sensitivity, specificity, and area under the curve (AUC) were calculated as measures of diagnostic accuracy. Out of the tested biomarkers, urinary transferrin, urinary Retinol binding protein (RBP) and serum osteopontin had the best diagnostic value for diabetic nephropathy presence based on the AUC value. The rest of the biomarkers had comparatively less or even no discriminative power. The urinary transferrin and RBP and serum osteopontin, had the best diagnostic value in type 2 diabetic patients at different stages of diabetic nephropathy. Further longitudinal prospective studies are needed to evaluate the predictive power of those markers for detecting diabetic nephropathy before any structural damage occurs.

## Introduction

Diabetic nephropathy is the most common cause of end stage renal disease (ESRD) that is associated with high rates of morbidity and mortality^1^. It is of utmost importance to emphasize the early identification and treatment of this chronic complication which would reduce the medical and economic burden associated with it^2^.

Although microalbuminuria is a widely used indicator for diabetic nephropathy, its diagnostic accuracy is limited by the fact that structural damage might precede albumin excretion^3^. Studies have also confirmed that microalbuminuria is not specific for the presence of diabetic nephropathy alone as non-diabetic patients with progressive chronic kidney disease may also develop microalbuminuria^4^. On the contrary, diabetic patients with microalbuminuria may also not progress to ESRD. Therefore, sensitive and specific biomarkers that can predict patient’s susceptibility to diabetic nephropathy are needed^5^.

Recently, there have been intensive research showing that different serum or urinary biomarkers have variable diagnostic accuracies in predicting glomerular or tubular kidney injury^6^. Most of the studies used one biomarker or focused on just one stage of diabetic nephropathy when assessing the diagnostic value to predict pathological changes that occur during the course of diabetic nephropathy^7, 8^. Therefore, studies assessing different biomarkers and their relation with different stages of diabetic nephropathy at molecular level may open a door for better understanding the disease leading to screening and prevention of this common chronic complication.

For the identification of novel diagnostic biomarkers that can be used to screen for diabetic nephropathy, literature review was conducted during the period from 1998 to 2014 to produce a list of serum/plasma and urinary biomarkers that were found to have a significant diagnostic value in diabetic nephropathy screening. A total of 18 serum and one plasma biomarker in addition to three urinary biomarkers were selected as shown in appendix 1. Therefore, the objective of the current study was to assess the diagnostic value of these biomarkers among type 2 diabetic patients at different stages of diabetic kidney disease (DKD) in a community facing diabetes epidemic.

## Results

### Patients’ characteristics

In this study, patients with diabetic nephropathy were significantly older with longer duration of diabetes than non-nephropathic patients, although the two groups were well matched in their height, weight, waist–hip circumference and waist hip ratio. There was no significant difference found between the two groups with respect to the percentage of males, family history of diabetes or renal disease. The presence of diabetic complications including; vasculopathy, retinopathy and other associated diseases including; hypertension and hyperlipidemia, was significantly more prevalent among nephropathic patients and especially among patients with marcoalbuminuria, as shown in Table 1.

**Table 1:** Baseline characteristics of the studied subjects according to their diabetic nephropathy stages.

Variables	All studied cohort				Nephropathy Sub-groups			
	All (467)	No nephropathy (200)	Nephropathy (267)	p-value	Microalbuminuria (184)	p-value	Macroalbuminuria (83)	p-value
Age (years) (±SD)	55.64 (±6.11)	54.93 (±5.93)	56.18 (±6.2)	0.029	56.47 (±5.83)	0.011	55.52 (±6.95)	0.471
DM Duration (years) (±SD)	18.26 (±5.59)	16.97 (±4.68)	19.22 (±6.02)	>0.001	19.08 (±5.99)	<0.001	19.54 (±6.13)	<0.001
Height (cm) (±SD)	160.91 (±9.21)	160.17 (±8.69)	161.46 (±9.57)	0.137	161.18 (±9.72)	0.283	162.06 (±9.25)	0.105
Weight (Kg) (±SD)	84.47 (±16.33)	84.28 (±15.19)	84.61 (±17.16)	0.830	84.93 (±17.15)	0.695	83.91 (±17.28)	0.855
BMI (Kg/m^2^) (±SD)	32.66 (±5.9)	32.90 (±5.69)	32.47 (±6.06)	0.438	32.69 (±5.88)	0.722	31.99 (±6.47)	0.238
Hip (cm) (±SD)	109.71 (±12.49)	109.60 (±11.75)	109.80 (±13.04)	0.869	110.13 (±12.35)	0.666	109.04 (±14.51)	0.732
Waist (cm) (±SD)	106.57 (±12.31)	105.80 (±12.03)	107.14 (12.5)	0.245	107.22 (±12.14)	0.252	106.98 (±13.34)	0.470
W/H ratio	0.97 (±.08)	0.97 (±.08)	0.98 (±.07)	0.120	0.98 (±.07)	0.279	0.98 (±.07)	0.090
SBP (mmHg) (±SD)	137.71 (±20.28)	130.27 (±16.97)	143.29 (±20.79)	<0.001	140.02 (±19.4)	<0.001	150.55 (±22.02)	<0.001
DBP (mmHg) (±SD)	74.01 (±11.2)	72.36 (9.95)	75.25 (±11.92)	0.004	74.60 (±11.27)	0.040	76.71 (±13.2)	0.008
eGFR ml/min 1.73 m^2^ body surface area (±SD)	69.88 (±20.82)	79.76 (±16.66)	61.65 (±20.38)	<0.001	66.50 (±19.25)	<0.001	50.17 (±18.38)	<0.001
ACR mg/g (±SD)	276.99 (±705.47)	10.44 (±10.23)	477.40 (±882.77)	<0.001	113.80 (±77.18)	<0.001	1293.28 (±1249.74)	<0.001
Male gender	212 (45.4%)	85 (42.5%)	127 (47.57%)	0.277	80 (43.48%)	0.847	47 (56.63%)	0.030
Family history of Diabetes	414 (88.65%)	180 (90%)	234 (87.64%)	0.426	160 (86.96%)	0.350	74 (89.16%)	0.831
Family history of Renal disease (%)	74 (15.85%)	32 (16%)	42 (15.73%)	0.975	32 (17.39%)	0.672	10 (12.05%)	0.401
Neuropathy (%)	236 (50.54%)	95 (47.5%)	141 (52.81%)	0.256	97 (52.72%)	0.307	44 (53.01%)	0.398
Retinopathy (%)	216 (46.25%)	62 (31%)	154 (57.68%)	<0.001	93 (50.54%)	<0.001	61 (73.49%)	<0.001
Vasculopathy (%)	83 (17.77%)	20 (10%)	63 (23.6%)	<0.001	39 (21.2%)	0.002	24 (28.92%)	<0.001
Hypertension (%)	329 (70.45%)	115 (57.5%)	214 (80.15%)	<0.001	141 (76.63%)	<0.001	73 (87.95%)	<0.001
Hyperlipidemia (%)	401 (85.87%)	164 (82%)	237 (88.76%)	0.029	160 (86.96%)	0.141	77 (92.77%)	0.020
Thyroid (%)	75 (16.06%)	41 (20.5%)	34 (12.73%)	0.036	27 (14.67%)	0.188	7 (8.43%)	0.016

ACR: Albumin creatinine ratio; BMI: body mass index; DM: diabetes mellitus; DBP: diastolic blood pressure; eGFR: estimated glumerular filtration rate; SBP: systolic blood pressure; W/H ratio: waist to hip ratio.

P value was calculated in reference to the patients without diabetic nephropathy.

### Distribution of biomarkers’ values according to ACR

**Figure 1:**
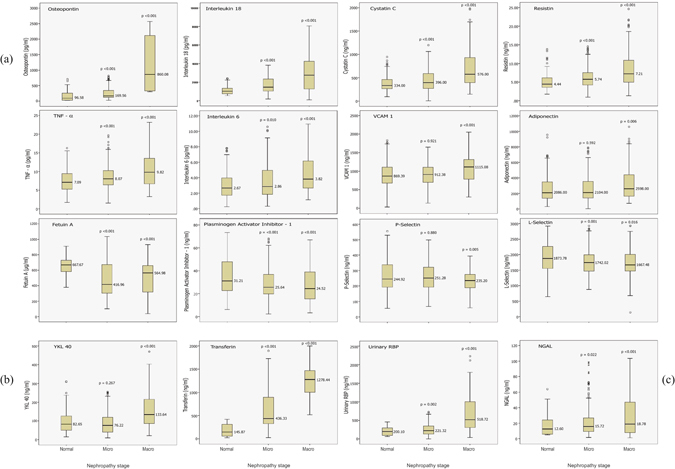
(**a**) Box plot values for the significant serum nephron-biomarkers according to different nephropathy stag. (**b**) Box plot values for the significant plasma nephron-biomarkers according to different nephropathy stages. (**c**) Box plot values for the significant urinary nephron-biomarkers according to different nephropathy stages. *P value compared with subjects with normal albumin excretion.

The effect of different stages of diabetic nephropathy on the values of each biomarker was represented by box plots,  as shown in Fig. 1a,b and c. The median value of 12 biomarkers, namely: urinary transferrin, serum osteopontin, urinary RBP, serum interleukin-18, serum cystatin C, serum resistin, plasma YKL-40, serum TNF-α, serum interleukin 6, serum VCAM-1, adiponectin and urinary NGAL had significantly increased in patients with microalbuminuria and marcoalbuminuria, except for plasma YKL-40, serum VCAM-1, and serum adiponectin among patients with microalbuminuria. Serum fetuin-A, PAI-1, P-selectin, and L-selectin had significantly decreased median values with diabetic nephropathy progression, while the median values of the remaining biomarkers did not significantly increase or decrease with nephropathy progression.

### Clinical and metabolic effect on biomarkers’ values

All the 22 biomarkers did not have any significant correlation with age or diabetes duration as shown in appendix 2. However, all biomarkers showed a significant positive correlation with ACR, except for serum E-selectin, CRP, and ICAM-1 which had a positive but non-significant correlation or MCP-1, P-selectin, L-selectin, PAI-1 and fetuin- A that had significant negative correlation with ACR. On the contrary, all the studied biomarkers had significant negative correlation with eGFR, except for some biomarkers,  as shown in Table 2.

**Table 2:** Correlation coefficient (r) between the 22 nephrobiomarkers and the studied patients’ parameters.

Biomarkers	Kidney function markers				Glycemic marker						Lipid markers						Clinical markers			
	ACR		eGFR		HOMA_IR		HbA1C		FPG		Triglycerides		Cholesterol		LDL		SBP		Weight	
	r	p	r	p	r	p	r	p	r	p	r	p	r	p	r	p	r	p	r	p
Transferrin (U) ng/ml	0.703	<0.001	−0.362	<0.001	0.18	0.001	0.169	<0.001	0.220	<0.001	0.245	<0.001	0.069	0.138	−0.024	0.611	0.312	<0.001	0.062	0.182
Osteopontin pg/ml	0.607	<0.001	−0.326	<0.001	0.174	0.002	0.212	<0.001	0.158	0.001	0.134	0.005	0.001	0.982	−0.079	0.098	0.282	<0.001	−0.013	0.783
RBP (U) ng/ml	0.229	<0.001	−0.142	0.003	0.002	0.972	0.079	0.096	0.025	0.595	0.005	0.917	0.009	0.853	−0.016	0.739	0.048	0.312	0.129	0.006
Interleukin−18 pg/ml	0.395	<0.001	−0.420	<0.001	0.132	0.018	0.187	<0.001	0.124	0.008	0.138	0.003	0.033	0.485	0.053	0.261	0.106	0.024	0.118	0.012
Cystatin-C ng/ml	0.332	<0.001	−0.492	<0.001	−0.023	0.682	0.059	0.219	−0.121	0.011	0.087	0.068	−0.038	0.431	−0.067	0.165	0.127	0.007	0.012	0.806
Resistin g/ml	0.339	<0.001	−0.344	<0.001	0.085	0.142	0.063	0.197	−0.037	0.444	0.057	0.24	−0.016	0.746	−0.125	0.01	0.106	0.029	0.077	0.113
YKL-40 (P) ng/ml	0.188	<0.001	−0.161	0.001	0.078	0.165	0.179	<0.001	0.049	0.305	0.092	0.053	−0.005	0.915	−0.051	0.288	0.115	0.015	0.086	0.070
TNF-α pg/ml	0.279	<0.001	−0.321	<0.001	0.283	<0.001	0.201	<0.001	0.061	0.193	0.14	0.003	0.037	0.424	0.05	0.287	0.137	0.003	0.009	0.849
Interleukin-6 pg/ml	0.226	<0.001	−0.198	<0.001	0.072	0.196	0.192	<0.001	−0.040	0.400	−0.059	0.212	−0.069	0.143	−0.063	0.185	0.166	<0.001	0.201	<0.001
VCAM-1 ng/ml	0.152	0.001	−0.194	<0.001	0.161	0.003	0.124	0.008	0.158	0.001	0.035	0.459	−0.014	0.767	−0.035	0.454	0.118	0.011	0.105	0.024
Adiponectin ng/ml	0.139	0.004	−0.122	0.013	−0.158	0.006	−0.087	0.071	−0.139	0.004	−0.196	<0.001	0.11	0.022	0.049	0.307	−0.013	0.793	−0.053	0.266
NGAL ng/ml	0.210	<0.001	−0.213	<0.001	0.120	0.029	0.093	0.045	0.012	0.792	0.080	0.084	0.012	0.797	0.014	0.758	0.102	0.027	0.036	0.437
Interleukin-1α pg/ml	0.201	<0.001	−0.224	<0.001	−0.09	0.135	−0.005	0.918	0.012	0.818	0.019	0.714	0.051	0.312	−0.03	0.551	−0.028	0.586	0.078	0.123
E-selectin ng/ml	0.002	0.965	−0.065	0.183	0.182	0.001	0.14	0.003	0.137	0.004	0.091	0.053	0	0.996	0.042	0.376	0.062	0.186	0.205	<0.001
CRP ng/ml	0.04	0.414	−0.032	0.521	0.12	0.038	0.228	<0.001	0.041	0.406	0.015	0.754	−0.047	0.336	−0.011	0.818	0.088	0.071	0.160	0.001
ICAM-1 ng/ml	0.017	0.724	−0.022	0.650	0.139	0.012	0.147	0.002	0.100	0.034	0.01	0.838	0.027	0.563	0.067	0.153	0.034	0.47	0.081	0.086
Leptin ng/ml	0.154	0.002	−0.128	0.011	0.106	0.066	0.127	0.009	−0.042	0.387	−0.034	0.488	0.143	0.003	0.07	0.151	0.095	0.051	0.169	0.001
MCP-1 pg/ml	−0.148	0.002	−0.035	0.475	−0.056	0.317	−0.143	0.002	−0.154	0.001	−0.058	0.22	−0.001	0.986	0.029	0.537	−0.054	0.254	0.054	0.247
P-selectin ng/ml	−0.092	0.049	−0.022	0.645	0.122	0.028	0.079	0.091	0.127	0.007	0.023	0.625	0.079	0.09	0.102	0.029	−0.042	0.366	0.099	0.034
L-selectin ng/ml	−0.142	0.002	0.057	0.237	−0.039	0.481	−0.023	0.63	0.002	0.971	−0.079	0.092	0.022	0.632	0.002	0.968	−0.05	0.285	0.096	0.039
PAI-1 ng/ml	−0.165	<0.001	0.140	0.004	−0.032	0.563	−0.042	0.372	−0.151	0.001	0.067	0.154	0.037	0.439	0.107	0.024	−0.085	0.073	−0.033	0.480
Fetuin-A µg/ml	−0.291	<0.001	0.061	0.204	−0.159	0.004	−0.141	0.002	−0.096	0.038	−0.079	0.087	0.015	0.752	0.056	0.23	−0.182	<0.001	−0.041	0.375

Urinary transferrin, serum osteopontin, interleukin 18, TNF-α, VCAM-1, urinary NGAL, serum E-selectin, CRP, ICAM-1, and P-selectin had significant positive correlation with increasing value HOMA-IR, while adiponectin and fetuin–A had significantly inverse correlation. All markers that were correlating with increasing value of HOMA-IR were positively significantly correlated with increasing value of HbA1c, except for P-selectin that was non-significantly correlated and YKL-40 and interleukin -6 that were significantly and positively correlated with HbA1c but not with HOMA-IR. Cystatin C, adiponectin, P-selectin and PAI-1 were significantly correlated with Fasting blood glucose but not with HbA1c, as shown in Table 3.

**Table 3:** Sensitivity, specificity and AUC for the 22 markers in detecting cases with microalbuminuria or macroalbuminuria.

Biomarkers	Microalbuminuria					Macroalbuminuria				
	Sensitivity	Specificity	AUC	PPV (%)	NPV (%)	Sensitivity	Specificity	AUC	PPV (%)	NPV (%)
ACR	0.995	0.955	0.995	99.5	99.5	1.00	0.995	1.00	98.8	100.0
Transferrin/CRE^1^	0.702	0.830	0.829	78.4	75.3	0.939	0.925	0.975	83.7	97.4
Osteopontin	0.888	0.547	0.692	63.7	84.6	1.00	0.80	0.938	68.6	100.0
RBP/CRE^1^	0.547	0.600	0.572	55.3	59.4	0.756	0.930	0.874	81.6	90.3
Interleukin-18	0.609	0.696	0.691	65.9	65.2	0.703	0.839	0.825	62.7	88.0
Cystatin C	0.630	0.526	0.588	54.8	61.0	0.722	0.684	0.770	48.7	85.5
Resistin	0.691	0.546	0.642	57.9	66.2	0.714	0.656	0.735	46.6	84.5
NGAL/CRE^1^	0.541	0.625	0.601	83.3	53.1	0.634	0.700	0.709	46.4	82.4
YKL-40^2^	0.386	0.554	0.455	44.2	49.8	0.628	0.674	0.698	43.8	81.8
TNF-α	0.776	0.407	0.602	54.6	66.4	0.605	0.638	0.688	40.5	79.9
Interleukin-6	0.457	0.651	0.560	54.1	57.2	0.627	0.626	0.680	41.6	79.7
VCAM-1	0.555	0.518	0.517	51.3	56.0	0.610	0.628	0.643	40.3	79.6
Adiponectin	0.423	0.628	0.511	51.4	53.9	0.573	0.628	0.606	38.1	78.7
Interleukin 1α	0.518	0.597	0.538	53.0	58.5	0.557	0.550	0.581	33.8	75.0
E-selectin	0.531	0.495	0.501	48.5	54.1	0.474	0.581	0.508	30.8	73.7
CRP	0.519	0.593	0.539	53.2	58.1	0.442	0.637	0.503	34.0	73.0
ICAM-1	0.534	0.490	0.504	48.5	53.9	0.519	0.551	0.502	31.5	74.1
Leptin	0.477	0.594	0.552	51.9	55.2	0.378	0.599	0.499	27.2	70.9
MCP-1	0.359	0.440	0.324	38.2	41.6	0.600	0.435	0.484	30.8	72.2
P-selectin	0.598	0.460	0.504	49.8	56.1	0.525	0.450	0.424	27.6	70.3
L-selectin	0.575	0.345	0.412	44.0	47.6	0.373	0.540	0.415	25.2	67.5
PAI-1	0.491	0.385	0.399	40.6	47.0	0.410	0.470	0.366	23.2	67.1
Fetuin A	0.219	0.705	0.283	40.4	49.6	0.205	0.645	0.289	19.3	66.2

All biomarkers were analyzed using blood serum, except for ^2^YKL-40 which was plasma in origin and ^1^transferrin, ^1^RBP, and ^1^NGAL which were urine samples presented as markers to creatinine ratio. PPV: positive predictive value; NPV: negative predictive value.

Area under the curve was classified according to the following: excellent (1.0-0.9); very good (0.9-0.8); good (0.8-0.7); sufficient (0.7-0.6) and bad or not useful (<0.6).

Urinary transferrin, osteopontin, interleukin-18, YKL-40, TNF-α and E-selectin had shown significantly increased values with higher level of triglyceride, while adiponectin had a significantly decreased value.

All the biomarkers were positively correlated with systolic blood pressure, either significantly or non-significantly, except for adiponectin, interleukin-1α, MCP-1, P-selectin, L-selectin, and PAI-1, that were non-significantly inversely correlated and fetuin-A that was significantly inversely correlated with systolic blood pressure, as shown in Table 3.

When looking at the patients’ body weights, urinary RBP, serum interleukin-18 and IL-6, VCAM-1, E-selectin, CRP, leptin, P-selectin and L-selectin, all had significant positive correlation, while the rest of the biomarkers were all non-significantly correlated with body weight either positively or negatively as shown in Table 2.

### Biomarkers sensitivity and specificity

Sensitivity, specificity and AUC were calculated to assess the significance of diagnostic value for each marker in the two diabetic nephropathy stages classified according to the ACR values, which had sensitivity, specificity and AUC of almost 1 in both groups when compared with patients with normal albumin excretion.

The studied biomarkers were arranged in descending order according to the AUC values only among the macroalbuminuria patients and were clustered into five groups as follows;

### Biomarkers with excellent diagnostic accuracy (AUC 1.0-0.9)

These biomarkers included urinary transferrin and RBP in addition to serum osteopontin. However their diagnostic accuracy was lower among patients with microalbuminuria, wherein it ranged from only sufficient for RBP to very good for transferrin.

### Biomarkers with very good diagnostic accuracy (AUC 0.9-0.8)

This group included only two biomarkers namely: interleukin-18 and cystatin C, but when looking at their diagnostic accuracy among microalbuminuria patients, it was found to be good for interleukin-18 and sufficient for cystatin-C.

### Biomarkers with good diagnostic accuracy (AUC 0.8-0.7)

Five biomarkers, namely; resistin, urinary NGAL, plasma YKL-4-, TNF-α and interleukin-6 were found to have good diagnostic accuracy among patients with microalbuminuria. All had sufficient diagnostic value among patients with microalbuminuria except for plasma YKL-40 which was found to be not useful.

### Biomarkers with sufficient diagnostic accuracy (AUC 0.7-0.6)

These biomarkers include VCAM-1, adiponectin and interleukin1-α, although they had no diagnostic value among patients with microalbuminuria.

### Biomarkers with no diagnostic value (AUC < 0.6)

These include: E-selectin, CRP, ICAM-1, leptin, MCP-1, P-selectin, L-selectin, PAI-1 and fetuin-A. None of these had any diagnostic value in microalbuminuria and macroalbumiuria patients.

## Discussion

Albuminuria or ACR has been considered for the last three decades as the golden standard diagnostic and prognostic biomarker for diabetic nephropathy onset and progression^9^. However, albuminuria lacks specificity for diagnosing disease progression when the urinary albumin excretion is <300 mg/24 h and also lacks sensitivity since diabetic nephropathy can frequently progress without an increase in albumin excretion. Therefore, it does not serve as an accurate surrogate endpoint for the progression of diabetic kidney disease.

The current study showed that all the 22 studied biomarkers had different levels of diagnostic accuracy ranging from excellent to very good to good according to AUC sensitivity and specificity values. All the biomarkers evaluated in this study were either markers of renal injury including markers of glomerular or tubular damage or markers of inflammation including pro-inflammatory cytokines, adhesion molecules, adipokines and Chemokines^10, 11^.

### Biomarkers of glomerular damage

Urinary transferrin has shown a gradual significant increase with diabetic nephropathy progression and had significant correlation positively with ACR, while negatively with eGFR. This positive correlation with ACR has also been observed in a cohort of Japanese type 2 diabetic patients as reported by Kazumi *et al*.^12^. In another study that was conducted earlier, increased urinary transferrin excretion was observed in 95% of patients with microalbuminuria and 100% of patients with macroalbuminuria^13^. These findings support the theory that transferrin is a sensitive and specific marker for the diagnosis of diabetic nephropathy. With respect to the diagnostic profile, transferrin/creatinine ratio had similar diagnostic accuracy of ACR for cases of macroalbuminuria, while it was close to ACR value in cases with microalbuminuria. Being a urinary marker, transferrin/creatinine ratio assessment is more practical and convenient. However, it could also reflect renal damages that are not related to diabetes i.e. primary glomerulonephritis^14^. The level of this marker was found to be increased with the presence of insulin resistance, poor glycemic control and elevated triglycerides, in addition to increased SBP. The correlation between urinary excretion and these clinical markers has been tested by Cheung *et al*., where the correlations were not significant except with SBP^13^. Additionally, urinary transferrin excretion has been found by Chelliah *et al*., in non-diabetic populations to be increased with hypertension^15^. Therefore, these factors, especially SBP should be taken in consideration when using this marker to screen for diabetic nephropathy.

### Biomarkers of Tubular damage

The pathophysiology of albuminuria and tubulointerstitial damage are considered to be intertwined, where on one hand the re-absorption of increased amount of protein from the tubular lumen, induces the pro-inflammatory and the profibrotic responses in tubular cells while on the other hand, the damage of the proximal renal tubules alone can lead to albumin leak and consequently albuminuria^13^.

RBP is a low–molecular weight protein that is filtered by the glomeruli and then reabsorbed and catalyzed by the proximal tubules^16^ and has shown very good diagnostic value in patients with macroalbuminuria, but to a lesser degree in subjects known to have microalbuminuria in the current study. There is an increased urinary excretion of RBP with the progression of diabetic nephropathy as observed in our study which was not affected by the metabolic status of the patients i.e., presence of hypertension or elevated lipid profile and is different from other studies that had shown significant increase in RBP level in patients with history of systolic hypertension.

Urinary NGAL, in this study, had a good diagnostic accuracy for marcoalbuminuria cases while sufficient diagnostic accuracy for microalbuminuria, although it has been reported to have better diagnostic profile for acute kidney injury among different populations^17^.

In the current study, urinary NGAL was significantly increasing in parallel with the deterioration of the disease and its urinary excretion had a statistically significant positive correlation with ACR. This finding was similar to what has been observed among a small cohort of Italian type 2 diabetic patients^15^ and Chinese patients^18^.

The current study showed a significant correlation between the urinary level of NGAL with insulin resistance, poor glycemic control and the presence of high SBP which is similar to what had been reported earlier in other ethnicities^19^.

Cystatin C is freely filtered by the renal glomeruli and metabolized by the proximal tubule, and could be detectable even before the appearance of microalbuminuria and the rise in serum creatinine^20^. Therefore cystatin C could be considered as a good candidate for the early detection of renal dysfunction in type 2 diabetic patients with normoalbuminuria. In this study, serum cystatin C has shown very good diagnostic profile for cases with marcoalbuminuria, while the value was only sufficient for cases with microalbuminuria. There are few studies that have assessed the correlation of cystatin C with clinical markers, although Heba *et al*., had demonstrated the increase of this biomarker with high SBP which is also similar to our findings^21^.

### Inflammatory markers

Although diabetic nephropathy is traditionally considered a nonimmune disease, there is an overwhelming evidence which indicates that immunologic and inflammatory mechanisms play a significant role in its development and progression. Individuals who progress to DN appear to display features of low grade inflammation for years before clinically detectable disease^22^.

The current study investigated the diagnostic profile of pro-inflammatory cytokines including; osteopontin, IL-1α, IL 6, IL18, TNF-α and CRP. In this study, the most important cytokine that were found to have significant diagnostic value were serum osteopontin^23^ and IL-18, which had shown an excellent diagnostic value for patients with macroalbuminuria and good diagnostic value for patients with microalbuminuria. These findings were consistent with what had been earlier reported among both type 1 and type 2 diabetic patients for osteopontin and in the study among Japanese type 2 diabetic patients for IL-18^24^. Henceforth, both of these markers can be considered as good markers for the progression of diabetic nephropathy as previously reported^25^.

TNF-α and IL-6 are two major pro-inflammatory cytokines that stimulate the acute phase response by triggering the production of other proteins such as CRP and α-1-acid glycoprotein (AGA)^26^. Both TNF-α and IL-6 had good diagnostic value for patients with macroalbuminuria but sufficient diagnostic accuracy for patients with microalbuminuria. In this study, IL-18, TNF- α and IL-6 had positive correlation with the degree of diabetes control and is similar to what had been observed in other studies^24–26^. On the other hand, Interleukin 1-α did not have any diagnostic value for patients with microalbuminuria but had only sufficient diagnostic value for marcoalbuminuria cases.

Adipokines including resistin and adiponectin are thought to have a deleterious effect on glomeruli and kidney functions through its pro-inflammatory effect^27^. In this study, resistin shows a good diagnostic value in micaroalbumiuria patients but sufficient value in microalbuminuria patients but with better values than adiponectin. The positive association between resistin and ACR in the current study was also observed among black and non-Hispanic diabetic patients^28^.

Adhesion molecules tested as biomarkers in this study included; VCAM-1, ICAM-1 and selectins. All these markers did not demonstrate any diagnostic value for both microalbuminuria and macroalbuminuria cases, except for VCAM-1 which had sufficient diagnostic value for cases with macroalbuminuria which was in accordance with the findings of Clausen *et al*. among type 1 diabetic patients, wherein the plasma level of VCAM-1 was elevated only in patients with overt diabetic nephropathy, but not the microalbuminuria patients^29^.

On the contrary, Plasma YKL-40 which is an endothelial dysfunction marker, has shown good diagnostic value only among patients with marcoalbuminuria and it had significant positive correlation with HbA1c, triglycerides and SBP but not with glycemic markers, or lipid markers as reported earlier^30^.

The rest of the studied markers in this study did not demonstrate any diagnostic value for both microalbuminuria and macralbuminuria cases.

The current study is limited by being a cross-sectional study design which only provides the basis for associations and not causality. Another limitation of the current study was non adjustment for hypertension and hyperlipidaemia as confounding factors, however, such adjustment is difficult since diabetic nephropathy would predispose for hypertension and hyperlipidaemia. On the other hand, this study draws its strength from assessing the diagnostic values of a large number of various biomarkers in different stages of diabetic nephropathy.

It is concluded from this study that urinary transferrin, urinary RBP and serum osteopontin had the best diagnostic value and needs to be further investigated with longitudinal prospective studies to evaluate the predictive power of these markers for diabetic nephropathy before any structural damage occurs. Interleukin-18 and cystatin C had very good diagnostic value, especially for cases with marcoalbuminuria, while resistin, NGAL, YKL-40, TNF-α and interleukin-6 had comparatively less discriminative power. VCAM-, IL-1α and adiponectin had sufficient diagnostic value for marcoalbuminuria cases. The rest of the markers did not have any diagnostic value.

## Methods

### Study design and setting

This is a cross-sectional hospital based study. Saudi type 2 diabetic patients aged between 35 to 70 years with diabetes duration of more than or equal 10 years were recruited from five general hospitals in Riyadh, according to the American Diabetes Association (ADA) criteria^31^ in a convenience series manner during the period from April 1^st^ 2014 till June 18^th^ 2015.

**Figure 2:**
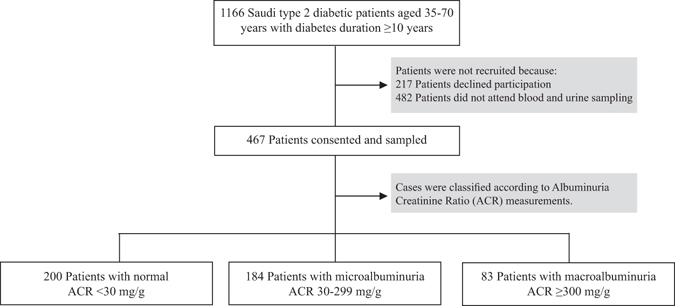
Study flow chart for case selection and recruitment.

Out of 6560 screened diabetic patients, only 1166 patients were eligible to participate in this study according to the abovementioned inclusion criteria, while those excluded were current smokers, pregnant, suffering from other causes of renal impairment or having ESRD. Out of the total eligible patients, 217 patients declined to participate and 482 patients did not attend blood and urine sampling visits. As a result, 467 patients were recruited for this study as shown in Fig. 2.

### Case identification

Patients were categorized into three groups depending on their urinary albumin excretion, evaluated as albumin/creatinine ratio (ACR) (mg/g creatinine). The first group included 200 diabetic patients withnormal albumin excretion (ACR <30 mg/g), while the second group consisted of 184 microalbuminuria patients (ACR between 30–299 mg/g), and the third group included 83 marcoalbuminuria cases (ACR ≥300 mg/g). Patients in both second and third groups were considered as patients with diabetic nephropathy. All the cases had eGFR >30 ml/min 1.73 m^2^ body surface area.

### Clinical covariates

Clinical covariates, that included blood pressure, height, weight, and waist to hip ratio in addition to history of neuropathy, retinopathy and vasculopathy as well as history of hypertension, hyperlipidemia and thyroid disease were obtained during the clinical visit.

### Biomarkers and metabolic assessment

After over-night fasting, cubital venous blood sample was collected from each patient for HbA1c and CBC, Lipid profile (total cholesterol, Low-density lipoprotein (LDL), High-density lipoprotein (HDL), triglycerides,) fasting glucose profile and kidney profile (urea and creatinine, uric acid). Assessments were performed through RX Daytona clinical chemistry analyzer by Randox (UK). Urinary creatinine and albumin were assessed using Rx Daytona clinical chemistry analyzer (UK). Insulin resistance [Homeostatic model assessment (HOMA) insulin resistance (IR) (HOMA-IR)] was calculated using the software HOMA 2 calculator (Oxford HOMA)^32^.

Patients were asked to provide two blood samples each for plasma and for serum extraction. Patients were also requested to provide fresh 10cc urine sample and these samples were immediately transferred to the central laboratory at the strategic center for diabetes research.

Most of the serum biomarkers were analyzed using biochip assay methodology with three arrays were performed; Metabolic Syndrome Array I (C-peptide, Insulin, Interleukin-1 alpha (IL-1α) Interleukin-6 (IL-6), Leptin, plasminogen Activator Inhibitor-1 (PAI-1), Tumor necrosis factor alpha (TNF-α) and Resistin), Metabolic syndrome array II (Adiponectin, C-Reactive Protein (CRP) and Cystatin C) and adhesion molecules array (Intercellular Adhesion Molecule-1 (ICAM-1) and Vascular Cell Adhesion Molecule-1 (VCAM-1) and E-Selectin, L-Selectin, P-Selectin). Serum Monocyte chemoattractant protein-1 (MCP-1), Osteopontin IL-18, Fetuin-A and plasma Human chitinase 3-like 1 (YKL-40) were not available in biochip array, therefore they were measured by enzyme-linked immunosorbent assay (ELISA) based Kit (Abcam, Cambridge, USA and R&D Kits UK).

The three urine biomarkers including urinary Transferrin, Retinol binding protein (RBP) and Lipocalin-2 (NGAL) levels were measured with enzyme-linked immunosorbent assay (ELISA) using commercially available standard kits (Abcam, Cambridge, USA).

### Statistical analysis

Data were analyzed using SPSS statistical package version 21. Continuous variables were expressed as mean ± standard deviation, and categorical variables were expressed as percentages. *t*-test was used for continuous variables and chi square test for categorical variables. The distribution of variables was assessed by examining the frequency histograms and the normal plots, and by using the Shapiro-Wilk test. Box plot was used as a visually - summarize tool and to compare the distributions of data for different groups. Sensitivity, specificity, and area under the curve (AUC) were calculated as measures of diagnostic accuracy. Receiver operating characteristic (ROC) curves were used to calculate the area under the curve (AUC). A perfect test has an area under the ROC curve of 1.0 and a faulty test has a value of ≤0.5. The Pearson correlation coefficient (r) was used to measure the strength and direction of a linear relationship between the 22 nephro-biomarkers and the studied patients’ parameters. The value of r is always between +1 and −1. The closer the correlation to 1, the stronger will be the relationship. A correlation of 0.0 indicates the absence of a relationship. A p-value of less than 0.05 was used as a level of significance.

### Disclosure

All authors declared no competing interest. All views, scientific finding, conclusions, and recommendations mentioned in the study represent the sole opinion of the research team and do not in any way reflect KACST views.

### Ethics approval and consent to participate

This study was reviewed and approved by the institutional review board at college of medicine, Kind Saud University, Research project No. E-13-1010. Informed consent forms were obtained from all the study participants and the study was conducted in accordance to declarations of Helsinki.

## Electronic supplementary material

Supplementary files
