# Supplementary material for: Assessment of the diagnostic value of different biomarkers in relation to various stages of diabetic nephropathy in type 2 diabetic patients
Source: Sci Rep. 2017 Jun 2;7:2684. doi: 10.1038/s41598-017-02421-9 (PMC5457399; doi:10.1038/s41598-017-02421-9)
Supplement: Supplementary file 1 — Supplementary files [file 41598_2017_2421_MOESM1_ESM.pdf]

Assessment of the diagnostic value of different biomarkers in relation to various stages of diabetic nephropathy in type 2 diabetic patients.

Khalid Al-Rubeaan, FRCPC1*,

krubeaan@dsrcenter.org

Khalid Siddiqui, PhD2,

Mohammed A Al–Ghonaim, FRCPC3,

Malghonaim@ksu.edu.sa

Amira M Youssef, B.Pharm4,

amagdi@ksu.edu.sa

Ahmed H. Al-Sharqawi, BSc1

ahmedh_1967@yahoo.com

Dhekra AlNaqeeb, MRes5

dlanaqeeb@ksu.edu.sa

1University Diabetes Center, Collage of medicine, King Saud University, Riyadh, PO Box 18397, Saudi Arabia

2Strategic Center for Diabetes Research, King Saud University, Riyadh, PO Box 245, Saudi Arabia.

3 Collage of medicine, King Khalid Universe Hospital, King Saud University, Riyadh, PO Box 245, Saudi Arabia

4Registry Department, University Diabetes Center, King Saud University, Riyadh, PO Box 245, Saudi Arabia.

5Research Department, University Diabetes Center, King Saud University, PO Box 245, Riyadh, Saudi Arabia.

*Corresponding author:
Khalid Al-Rubeaan, MD, FRCP(C)
University Diabetes Center
King Saud University
PO Box 18397
Riyadh 11415

Supplementary 1: Correlation coefficient (r) between the 22 nephrobiomarkers and the studied patients' parameters

| | Age | | Duration of diabetes | |
|---|---|---|---|---|
| | r | p | r | p |
| Transferrin (U) | 0.012 | 0.797 | 0.115 | 0.013 |
| Osteopontin | 0.034 | 0.475 | 0.091 | 0.056 |
| RBP (U) | -0.050 | 0.291 | -0.029 | 0.539 |
| Interleukin-18 | -0.001 | 0.977 | 0.124 | 0.008 |
| Cystatin-C | 0.086 | 0.071 | 0.025 | 0.602 |
| Resistin | -0.004 | 0.930 | 0.084 | 0.083 |
| YKL-40 (P) | 0.082 | 0.084 | 0.058 | 0.225 |
| TNF-α | 0.087 | 0.061 | 0.068 | 0.146 |
| Interleukin-6 | 0.094 | 0.045 | 0.036 | 0.449 |
| VCAM-1 | 0.064 | 0.171 | 0.067 | 0.147 |
| Adiponectin | 0.060 | 0.213 | 0.033 | 0.491 |
| UNGAL | 0.043 | 0.351 | -0.035 | 0.457 |
| Interleukin-1α | -0.005 | 0.928 | 0.008 | 0.875 |
| E-selectin | -0.036 | 0.438 | -0.016 | 0.737 |
| CRP | -0.004 | 0.935 | -0.017 | 0.722 |
| ICAM-1 | -0.039 | 0.405 | 0.039 | 0.410 |
| Leptin | 0.026 | 0.595 | -0.018 | 0.717 |
| MCP-1 | 0.009 | 0.846 | -0.019 | 0.689 |
| P-selectin | -0.023 | 0.625 | 0.008 | 0.865 |
| L-selectin | -0.148 | 0.001 | -0.044 | 0.342 |
| PAI-1 | -0.077 | 0.102 | -0.128 | 0.007 |
| Fetuin-A | 0.006 | 0.889 | -0.052 | 0.264 |

Supplementary 2. List of the selected biomarkers for diabetic nephropathy

| Markers | Functions | Study outcome | References |
|---|---|---|---|
| Serum Interleukin-6 (IL-6) | A pleiotropic cytokine which stimulates mesangial cell proliferation and enhances endothelial permeability. | Involve in inflammation and development of kidney injury in DM. A positive correlation is reported with IL-6 and glomerular basement membrane(r=0.25, p<0.05). | • Dalla Vestra M, Mussap M, Gallina P, et al. Acute-phase markers of inflammation and glomerular structure in patients with type 2 diabetes. J Am Soc Nephrol. 2005;16 Suppl 1:S78-82. |
| Serum Interleukin-1a (IL-1a) | Involved in the development of abnormalities in intraglomerular hemodynamics related to prostaglandin synthesis by mesangial cells. | Increased expression has been reported in experimental model of diabetes. | • Navarro-González JF, Mora-Fernández C. The role of inflammatory cytokines in diabetic nephropathy. J Am Soc Nephrol 2008; **19**: 433–442. |
| Serum Interleukin-18 (IL-18) | IL-18 is a potent inflammatory cytokine that induces IFN, which in turn induces functional chemokine receptor expression in human mesangial cells. | Elevated levels of IL-18 are a predictor of future renal dysfunction in type 2 diabetic patients with normoalbuminuria (OR (95% CI: 3.6(1.2-10.4)). | • Navarro-González JF, Mora-Fernández C. The brole of inflammatory cytokines in diabetic nephropathy. J Am Soc Nephrol 2008; **19**: 433–442.<br>• S. Araki, M. Haneda, D. Koya, T. Sugimoto, K. Isshiki, M. Chin-Kanasaki, et al., Predictive impact of elevated serum level of IL-18 for early renal dysfunction in type 2 diabetes: an observational follow-up study, Diabetologia 50 (2007) 867–873. |
| Serum Monocyte chemoattractant protein-1 (MCP-1) | MCP-1 promotes the transmigration of circulating monocytes into tissues and infiltration and activation of monocytes have been implicated in the development of glomerular injury. | MCP-1 serum concentrations manifest positive association with albuminuria and negative association with eGFR in T2D and detected significant positive association with MCP1 and urine ACR after logarithmic transformation (parameter estimate 0.0021, P=0.04). | • Mariana Murea , Thomas C Register , Jasmin Divers , Donald W Bowden , J Jeffrey Carr , Caresse R Hightower , Jianzhao Xu , S Carrie Smith , Keith A Hruska , Carl D Langefeld and Barry Freedman Relationships between serum MCP-1 and subclinical kidney disease: African American Diabetes Heart Study Murea et al. BMC Nephrology 2012, 13:148. |
| Serum Tumour necrosis factor alfa (TNF-α) | TNFa is a pleiotropic cytokine that plays an essential role in mediating inflammatory processes. TNF-α-induced podocytopathy. | TNF-α in diabetic patients with microalbuminuria or clinical albuminuria were significantly increased as compared with those without albuminuria (TNF-α, 3.20 ± 0.41 pg/mL *v* 1.94 ± 0.18 pg/mL). | • Moriwaki Y, Yamamoto T, Shibutani Y, Aoki E, Tsutsumi Z, Takahashi S, Okamura H, Koga M, Fukuchi M, Hada T. Elevated levels of interleukin-18 and tumor necrosis factor-α in serum of patients with type 2 diabetes mellitus: Relationship with diabetic nephropathy Metabolism. 2003 May;52(5):605-8. |
| Serum L-selectin | L selectins are leukocyte adhesion molecules, which mediate leukocyte infiltration into the inflammatory lesions. | The interaction between L-selectin and their major binding protein sulfatide plays a critical role in monocyte infiltration into the kidney interstitium; and involved in pathogenesis of DN. | • Ogawa D, Shikata K, Honke K, *et al*. Cerebroside sulfotransferase deficiency ameliorates L-selectin-dependent monocyte infiltration in the kidney after ureteral obstruction. J Biol |

| | | | |
|---|---|---|---|
| | | | Chem 2004; **16**: 2085–2090. |
| Serum E-selectin | E-selectin is expressed on endothelium and contributes to monocyte adhesion to endothelium | Expression of E-selectin is upregulated in the peritubular capillaries and is correlated with the number of infiltrating macrophages in the interstitium of patients with diabetic nephropathy. | • Narumi S, Onozato ML, Tojo A, *et al* Tissue-specific induction of E-selectin in glomeruli is augmented following diabetes mellitus. Nephron 2001; 89: 161–171. |
| Serum P-selectin | P-selectin that is expressed on endothelium or released from platelets contributes to neutrophils or monocytes rolling on the endothelium. | Expression of P- selectin are significantly increased in the glomeruli and the interstitium of patients with diabetic nephropathy as compared with those with other glomerular diseases. | • K. Hirata, K. Shikata, M.Matsuda, K.Akiyama, H. Sugimoto, M.Kushiro, H.Makino (1998) Increased expression of selectins in kidneys of patients with diabetic nephropathy, Diabetologia 41: 185-192. |
| Serum Fetuin A | A circulating calcium-regulatory glycoprotein synthesized in the liver .It act as an endogeneous inhibitor of the insulin receptor tyrosine kinase. | Serum Fetuin A could be responsible for the development and progression of accelerated nephropathy especially with uncontrolled diabetes. Albumin excretion were significantly and inversely associated with serum fetuin-A levels (r = -0.56, P<0.001). | • Ayman Ramadan, 1Amira Shoukry, Mabrouk I. Ismail and Maher Borai. Serum Fetuin-A Levels in Type 2 Diabetes Patients with Early Diabetic Nephropathy: It's Relation to Diabetes Control Journal of American Science 2011;7(5):759-765]. (ISSN: 1545-1003). |
| Serum Osteopontin | Expressed in glomerular cells like mesangial cells, podocytes, and endothelial cells and one of the key pathophysiologic contributor in DN. | Serum OPN is a strong predictor of incipient diabetic nephropathy, a first-ever CVD event, and all-cause mortality in patients with T1D. Cox regression analysis for incident microalbuminuria (HR 1.03 (1.01–1.05) p=0.03). | • Gordin D, Forsblom C, Panduru NM, Thomas MC, Bjerre M, Soro-Paavonen A, Tolonen N, Sandholm N, Flyvbjerg A, Harjutsalo V, Groop PH, FinnDiane Study Grou. Osteopontin is a strong predictor of incipient diabetic nephropathy, cardiovascular disease, and all-cause mortality in patients with type 1 diabetes. Diabetes Care. 2014 Sep;37(9):2593-600. |
| Serum Plasminogen activator inhibitor-1 (PAI-1) | Plasmin mediates the degradation of the extracellular matrix either by itself or in conjunction with matrix metalloproteinases. | PAI-1 contributes to diabetic nephropathy by regulating TGF-beta and renal ECM production. | • Nicholas SB, Aguiniga E, Ren Y, Kim J, Wong J, Govindarajan N, Noda M, Wang W, Kawano Y, Collins A, Hsueh WA. Plasminogen activator inhibitor-1 deficiency retards diabetic nephropathy. Kidney Int. 2005 Apr;67(4):1297-307. |
| Serum Cystatin C | Cystatin C has a low molecular weight (approximately 13.3 kilodaltons), and it is removed from the bloodstream by glomerular filtration in the kidneys. If kidney function and glomerular filtration rate | The cystatin C levels of serum could be useful markers for renal dysfunction in type 2 diabetic patients with normoalbuminuria. (AUC -0.906 (95% CI, 0.865- 0.947) with a cutoff value - 1.06, sensitivity-81.0%; specificity-87.1%). | • Yun Kyung Jeon, Mi Ra Kim, Jung Eun Huh, Ji Young Mok, Sang Heon Song, Sang Soo Kim, Bo Hyun Kim, Soo Hyoung Lee, Yong Ki Kim,and In Joo Kim Cystatin C as an Early Biomarker of Nephropathy in Patients with Type 2 Diabetes. J Korean Med Sci. 2011 Feb; 26(2): 258–263. |

| | | | |
|---|---|---|---|
| | decline, the blood levels of cystatin C rise. | | |
| Serum Resistin | One of the major cytokines secreted by adipose tissue (adipokines), it is a cysteine-rich protein that is primarily secreted by the macrophages embedded in the adipose tissue and has been linked to insulin resistance and low-grade inflammation. | There is an independent association between serum resistin and GFR among patients with T2D. (OR 1.33 (1.16–1.53) <0.001).). | • Moreno LO, Salvemini L, Mendonca C, Copetti M, De Bonis C, De Cosmo S, et al. (2015) Serum Resistin and Glomerular Filtration Rate in Patients with Type 2 Diabetes. PLoS ONE 10(3): e0119529 . |
| Serum Leptin | Leptin may mediate glomerular hypertrophy and sclerosis by stimulating glomerular endothelial and mesangial cell proliferation. | Serum leptin levels are elevated in type 2 diabetic patients with microalbuminuria and macroalbuminuria suggesting that renal leptin degradation is impaired in the early stages of renal disease and this impairment increases with the progression of renal disease (P>0.01). | • Mohammad A. kopeisy , Haitham A.Azeem and Salwa I.Wasfy Evaluation of leptin levels in serum of patients with non insulin dependant diabetic nephropathy AAMJ, Vol.9, N. 3, September, 2011. |
| Serum Adiponectin | A hormone that is secreted exclusively from adipocytes, reported to have insulin-sensitizing, anti-inflammatory, and anti-atherogenic properties | An inverse and independent association between serum adiponectin and GFR among Italian patients with T2D (OR 1.36 (1.16–1.60) <0.0001). | • Moreno LO, Salvemini L, Mendonca C, Copetti M, De Bonis C, De Cosmo S, et al. (2015) Serum Adiponectin and Glomerular Filtration Rate in Patients with Type 2 Diabetes. PLoS ONE 10(3): e0119529 . |
| Serum Intercellular Adhesion Molecule 1 (ICAM 1) | An adhesion molecules responsible for the tight adhesion of leukocytes to the endothelium and studies have postulated that ICAM-1 may play a role in the pathogenesis of diabetic nephropathy. | Serum ICAM 1 level have an important role in predicting the progression of diabetic kidney disease in albuminuric patients(uACR (r= 0.31, p=0.003). | • Alina Ramona Potra1 , Ina Maria Kacso, Ioana Cosmina Bondor , Mirela Gherman-Căprioară . The predictive value of serum intercellular adhesion molecule 1 for the progression of diabetic kidney disease in type 2 diabetic patients. Revista Română de Medicină de Laborator Vol. 21, Nr. 4/4, Decembrie 2013. |
| Serum Vascular cell adhesion protein 1 (VCAM1) | An inflammatory cytokines that is involved in leukocyte-adhesion recruitment into the kidney during inflammation and its expression was detected in the vascular endothelium and infiltrating cells in the kidney of diabetic patients | Circulating soluble adhesion molecules were correlated with albuminuria, we found a significant correlation between VCAM-1 levels and 24-hour urinary albumin excretion ($r = 0.4$, $p < 0.02$). | • Rubio-Guerra A.F. Vargas-Robles H. Lozano Nuevo J.J. Escalante-Acosta B.A Correlation between Circulating Adhesion Molecule Levels and Albuminuria in Type-2 Diabetic Hypertensive Patients Kidney Blood Press Res 2009;32:106–109. |

| | | | |
|---|---|---|---|
| Serum C - Reactive protein (CRP) | C-reactive protein (CRP) is an annular (ring-shaped), pentameric protein found in blood plasma, whose levels rise in response to inflammation. It is an acute-phase protein of hepatic origin that increases following interleukin-6 secretion by macrophages and T cells. | Serum hs-CRP levels, independent of possible confounders, were associated with a subsequent risk of developing diabetic nephropathy in type 2 diabetic patients. Association between serum hs-CRP concentration and shift from normoalbuminuria to microalbuminuria (HR 1.57 (1.22–2.03) p<0.001). | • Hayashino Y, Mashitani T, Tsujii S, Ishii H; Diabetes Distress and Care Registry at Tenri Study Group. Serum high-sensitivity C-reactive protein levels are associated with high risk of development, not progression, of diabetic nephropathy among Japanese type 2 diabetic patients: a prospective cohort study (Diabetes Distress and Care Registry at Tenri [DDCRT7]). Diabetes Care. 2014 37(11): 2947-52. |
| Plasma chitinase-3-like-1 (YKL-40) | A glycoprotein involved in inflammation and endothelial dysfunction. | Plasma YKL-40, was an independent factor associated with albuminuria in early stage of nephropathy in type 2 diabetes. plasma YKL-40 was significantly correlated with albuminuria (r=0.359; P=0.001). | • Lee JH, Kim SS, Kim IJ, Song SH, Kim YK, In Kim J, Jeon YK, Kim BH, Kwak IS. Clinical implication of plasma and urine YKL-40, as a proinflammatory biomarker, on early stage of nephropathy in type 2 diabetic patients. J Diabetes Complications. 2012 Jul-Aug;26(4):308-12. |
| Urinary Tranferrin | Transferrin has been proposed as a mediator of tubular toxicity because the reabsorption of transferrin results in the release of reactive iron in proximal tubular cells, promoting the formation of hydroxyl radicals | A significant correlations between the urinary excretion of transferrin and the degree of interstitial fibrosis (*r*=50.637, *P*<0.0001), tubular atrophy (*r*=50.468, *P*,0.01), and interstitial in- flammatory cell infiltration (*r*=50.503, *P*<0.001). | • Kanauchi M, Akai Y, Hashimoto T. Transferrinuria in type 2 diabetic patients with early nephropathy and tubulointerstitial injury. Eur J Intern Med. 2002 May;13(3):190-193. |
| Urinary Neutrophil Gelatinase-Associated Lipocalin (NGAL) | These proteins play a role in binding and transporting small hydrophobic molecules, apoptosis and immune regulation and play a renoprotective role as a mediator of tubular cell proliferation. | UNGAL can be early elevated even before early signs of glomerular injury detected by microalbuminuria ( AUC-0.87 ,sensitivity-80.4% and specificity-80% ). | • Abeer A. Al-Refai, Safaa I. Tayel, Ahmed Ragheb, Ashraf G. Dala, Ahmed Zahran. Urinary Neutrophil Gelatinase Associated Lipocalin as a Marker of Tubular Damage in Type 2 Diabetic Patients with and without Albuminuria Open Journal of Nephrology, 2 014, 4, 37-46. |
| Urinary Retinol binding protein (RBP) | Retinol binding protein (RBP) low molecular weight protein (21 kDa) which is freely filtered at the glomerulus and then almost completely reabsorbed in the proximal tubule. | Urinary RBP is independently related to the risk of CKD progression in patients with macroalbuminuric Diabetic nephropathy (OR 11.6; 95% CI 2.7–49.2, p=0.001 for log RBP). | • Titan SM, Vieira JM Jr, Dominguez WV, Moreira SR, Pereira AB, Barros RT, Zatz R. Urinary MCP-1 and RBP: independent predictors of renal outcome in macroalbuminuric diabetic nephropathy. J Diabetes Complications. 2012 Nov-Dec;26(6):546-53. |
